# Corrigendum

**DOI:** 10.1111/mpp.12992

**Published:** 2020-10-12

**Cui, X., Wei, Y., Wang, Y.H., Li, J., Wong, F.L., Zheng, Y.J. *et al*.** (2015) Proteins interacting with mitochondrial ATP‐dependent Lon protease (MAP1) in *Magnaporthe oryzae* are involved in rice blast disease. *Molecular Plant Pathology.* 16, 847–859.

The authors have been made aware of an error in Figure 2b for this paper in which some parts of the images in the figure were inadvertently duplicated.

**FIGURE 2:**
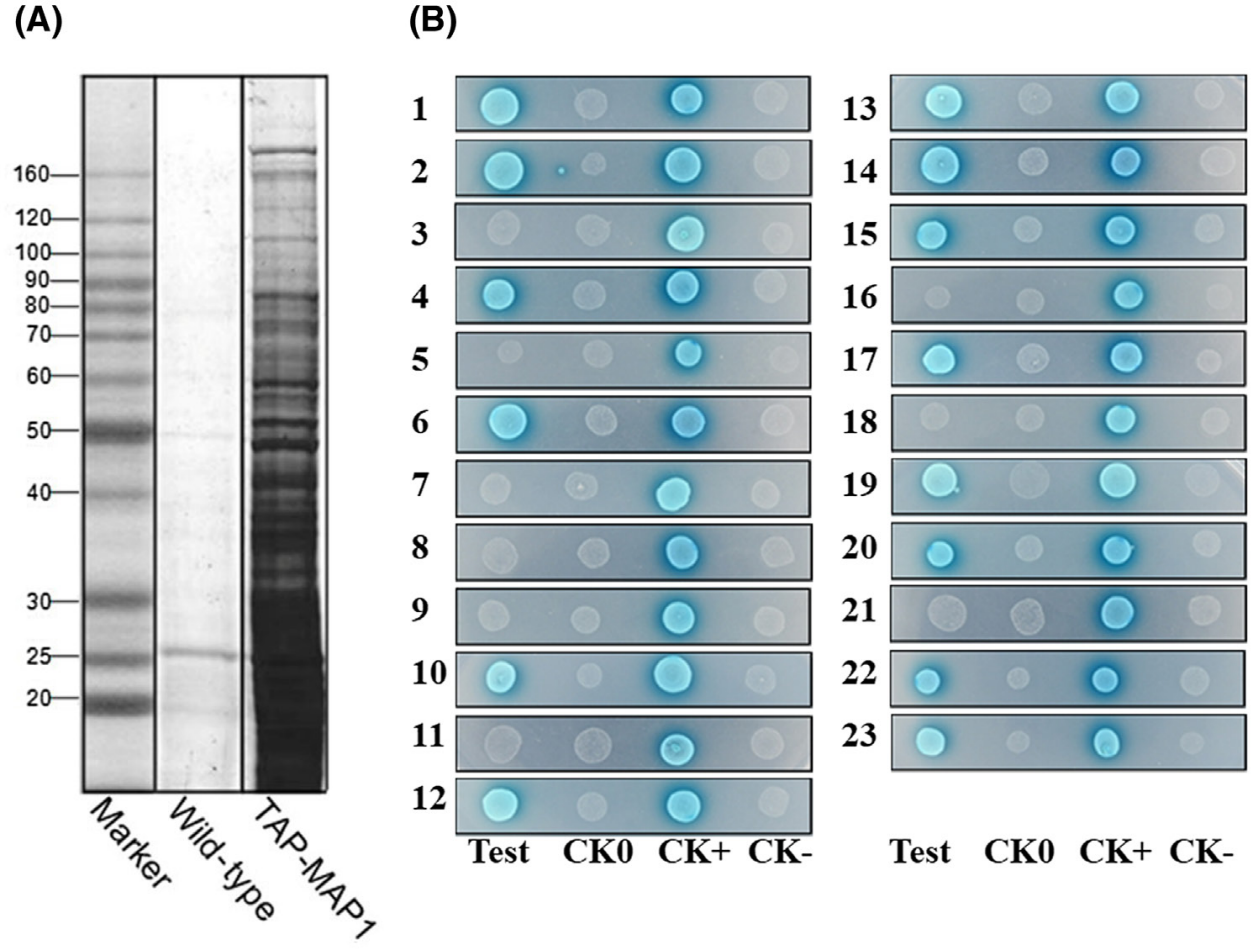
Identification of MAP1‐interacting proteins. (A) Tandem affinity purification (TAP) of MAP1‐interacting proteins. Elution fractions were analysed using sodium dodecylsulfate‐polyacrylamide gel electrophoresis (SDS‐PAGE) with Coomassie blue staining. (B) Yeast two‐hybrid assay. Test, interaction between MAP1 and each interacting candidate; CK0, self‐activation control; CK+, positive control; CK–, negative control. Each interacting protein is represented by a number (1–23) and is listed in Table 1.

The authors have repeated the experiment in strict accordance with the original description. Before performing the yeast‐two hybrid assay, they sequenced the *MAP1* gene contained in the bait vector, each of the 23 interacting genes contained in the prey vector, and matched sequences to those deposited in NCBI (https://www.ncbi.nlm.nih.gov/gene/). All tested constructs were confirmed to be correct, which were then cotransformed into AH109 (Clontech). The results demonstrated that 14 out of 23 candidates bound directly to MAP1 in yeast, including AnnA7, SHM, RanBP, C3HC, CGT, C2H2, AOX, ECH1, Bud6p, PrePL, CHP1, TauD, MMT, and CHS6. The experimental results are therefore consistent with those originally published in the paper.

The updated Figure 2 is provided here.

